# The *Alternaria alternata* Mycotoxin Alternariol Suppresses Lipopolysaccharide-Induced Inflammation

**DOI:** 10.3390/ijms18071577

**Published:** 2017-07-20

**Authors:** Shivani Grover, Christopher B. Lawrence

**Affiliations:** Department of Biological Sciences, Virginia Tech, Blacksburg, VA 24061, USA; shgrover@vt.edu

**Keywords:** *Alternaria alternata*, alternariol, innate immunity, immunosuppression

## Abstract

The *Alternaria* mycotoxins alternariol (AOH) and alternariol monomethyl ether (AME) have been shown to possess genotoxic and cytotoxic properties. In this study, the ability of AOH and AME to modulate innate immunity in the human bronchial epithelial cell line (BEAS-2B) and mouse macrophage cell line (RAW264.7) were investigated. During these studies, it was discovered that AOH and to a lesser extent AME potently suppressed lipopolysaccharide (LPS)-induced innate immune responses in a dose-dependent manner. Treatment of BEAS-2B cells with AOH resulted in morphological changes including a detached pattern of growth as well as elongated arms. AOH/AME-related immune suppression and morphological changes were linked to the ability of these mycotoxins to cause cell cycle arrest at the G2/M phase. This model was also used to investigate the AOH/AME mechanism of immune suppression in relation to aryl hydrocarbon receptor (AhR). AhR was not found to be important for the immunosuppressive properties of AOH/AME, but appeared important for the low levels of cell death observed in BEAS-2B cells.

## 1. Introduction

The fungal genus *Alternaria* harbors many plant and human pathogens, saprophytes, and allergenic species, and has been shown to be a prolific producer of secondary metabolites [1]. *Alternaria* spores are ubiquitous, and exposure has been clinically associated with the development, onset, and exacerbation of allergic diseases such as allergic rhinitis, asthma, and chronic rhinosinusitis (CRS) [2,3,4,5,6,7]. Indeed, up to 70% of mold allergy patients have skin test reactivity to *Alternaria.* Over 10 allergen proteins have been described from *Alternaria*, however, the secreted major allergen, Alt a 1, produces a prolonged and intense IgE-mediated reaction in sensitized patients [3,4,5,6]. Despite the well-documented clinical relevance of proteinaceous allergens, no small molecules (secondary metabolites) from *Alternaria* have been studied in regard to lung epithelium, inflammation, and immune responses.

Fungal mycotoxins are products of their secondary metabolism that can often cause deleterious effects in vertebrates. These secondary metabolites belong to different chemical classes such as steroids quinones, pyrones, peptides, phenolics, and the fumonisin-like toxins. These toxins can enter the body through skin, mucous, airways, and ingestion. Constant exposure can lead to hypersensitivity and mycotoxicosis, leading to a potentially compromised immune system and the onset of other illnesses and infections (HIV, kidney and liver damage) [7,8]. However, of all the mycotoxins known, only a few are subject to regular monitoring of contamination and level intake such as aflatoxins from *Aspergillus* spp., and fumonisins, deoxyivalenol, zearlenone, and ochratoxin-A from other fungi like *Fusarium* spp. Legal authorities from both food and feed industries acknowledge the importance of detecting and quantifying mycotoxin levels and identifying the effects of their contamination [9].

Besides producing deleterious mycotoxins, fungi are also an important resource of potential beneficial compounds with therapeutic properties. Ever since the discovery of penicillin from *Penicillium notatum* in 1929, the importance of elucidating the potential of fungal secondary metabolites has been beyond question [10]. *Alternaria* metabolites have exhibited a variety of therapeutic and biological properties such as phytotoxicity, cytotoxicity, anti-HIV, anti-cancer, and anti-microbial properties, to name a few, all of which have generated considerable research interest worldwide. For example, porritoxin from *Alternaria porri* is a likely cancer chemo-preventive agent, and depudecin from *Alternaria brassicicola* is an inhibitor of histone deacetylase [11,12,13,14].

**Figure 1:**
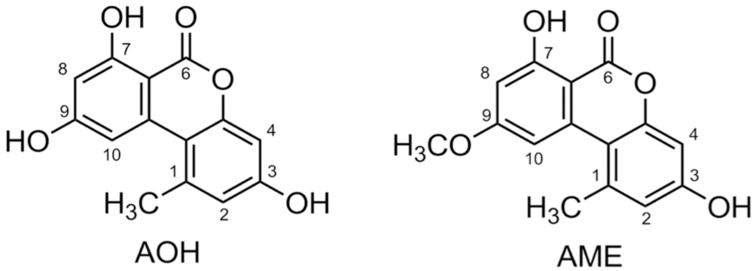
Chemical structure of alternariol (AOH) and alternariol monomethyl ether (AME).

The most well-studied deleterious *Alternaria* mycotoxins alternariol (AOH) and alternariol methyl ether (AME) have been detected in most foods and grains, often at high concentrations (Figure 1) [15]. Foods such as apples, apple products, mandarins, olives, pepper, tomatoes, oilseed, sunflower seeds, sorghum, wheat, edible oils, citrus fruits, melons, pears, prune nectar, raspberries, red currant, carrots, barley, oats, red wine, and lentils are known to be frequently contaminated with AOH/AME. The maximum levels reported are in the range of 1–103 μg/kg, with higher levels found in food products visibly rotted with *Alternaria* [15]. However, as of yet, no data concerning tissue levels of AOH exists in animals and humans [13,16].

AOH was shown to cause mutagenicity and cytotoxicity in Chinese hamster V79 cells [17,18]. AOH is also known to cause the formation of micronuclei (MN) in V79 and human endometrial adenocarcinoma cell line (Ishikawa cells) [17,18]. Treatment of AOH on murine macrophage cell line RAW 264.7 showed cytotoxicity and DNA strand breakage, as well as oxidative damage, cellular stress, and cell cycle arrest in the G2/M phase [18]. Human adenocarcinoma cells (HT29) treated with AOH indicated that the toxin modulates levels of reactive oxygen species (ROS) [19].

The aryl hydrocarbon receptor (AhR) is a ligand-activated transcription factor that often binds to environmental toxins and subsequently modulates the downstream expression of genes involved in detoxification and transport [20]. It has been studied in relation to various environmental contaminants such as the xenobiotic compound TCDD (2,3,7,8-tetrachlorodibenzo-p-dioxin) [21]. Binding of the AhR to the ligand causes the translocation of the complex to the nucleus and binding with AhR nucleus translocator (ARNT) [20]. The AhR-ARNT complex then binds to various xenobiotic response elements (XREs) and modulates the induction of genes, for instance the cytochrome P450 family [20]. AhR is a potential receptor for AOH and AME. The CYP450 family of genes, including the highly expressed CYP1A1, are a major target of the AhR-ARNT complex and often mediate toxin hydroxylation and further metabolism [21,22]. AOH has a planar structure that is similar to other AhR ligands and may be a substrate of CYP1A1. Further evidence of this was substantiated by the treatment of AOH on murine hepatoma cells, resulting in the differential expression of CYP1A1 in the presence and absence of activated and inactivated AhR [21,22]. This study is the first attempt at providing an experimental framework to investigate the immune-modulatory properties and potential clinical importance of *Alternaria* secondary metabolites, the mycotoxins alternariol (AOH) and alternariol methyl ether (AME).

## 2. Results

### 2.1. Alternariol Suppresses Innate Immune Responses in Human Lung Epithelial and Mouse Macrophage Cell Lines

We first sought to characterize the response of the mammalian innate immune system by quantifying cytokine and chemokine inflammatory markers upon AOH treatment on airway epithelial cells. We profiled cytokine interleukin-6 (IL6) in addition to chemokines interleukin-8 (IL8) and monocyte chemoattractant protein-1 (MCP-1/CCL2), which have been shown to be highly induced in many inflammatory diseases including sepsis [23].

We hypothesized initially that AOH/AME would have proinflammatory effects on cells. We evaluated the protein and mRNA levels of IL6 and IL8 after the treatment of bronchial epithelial cells (BEAS-2B) with AOH. Surprisingly, we found that AOH did not cause an increase in the protein levels of IL6 and IL8, but resulted in downregulation at the mRNA level after 6, 12, and 24 h of incubation. This data is summarized in Appendix A, Table A1. Because AOH may not be able to induce the primary inflammation markers (IL6, IL8, MCP-1/CCL2); we also examined other cytokine and chemokine markers that might be stimulated by AOH. We subsequently analyzed the protein levels of thymic stromal lymphopoietin (TSLP), tumor necrosis factor α (TNF-α), interleukin-1 β (IL-1β), interleukin-10 (IL-10), and transforming growth factor β (TGF-β), but no induction was observed. Primer sequences for gene expression analyses performed throughout the study are shown in Appendix A, Table A2.

**Figure 2:**
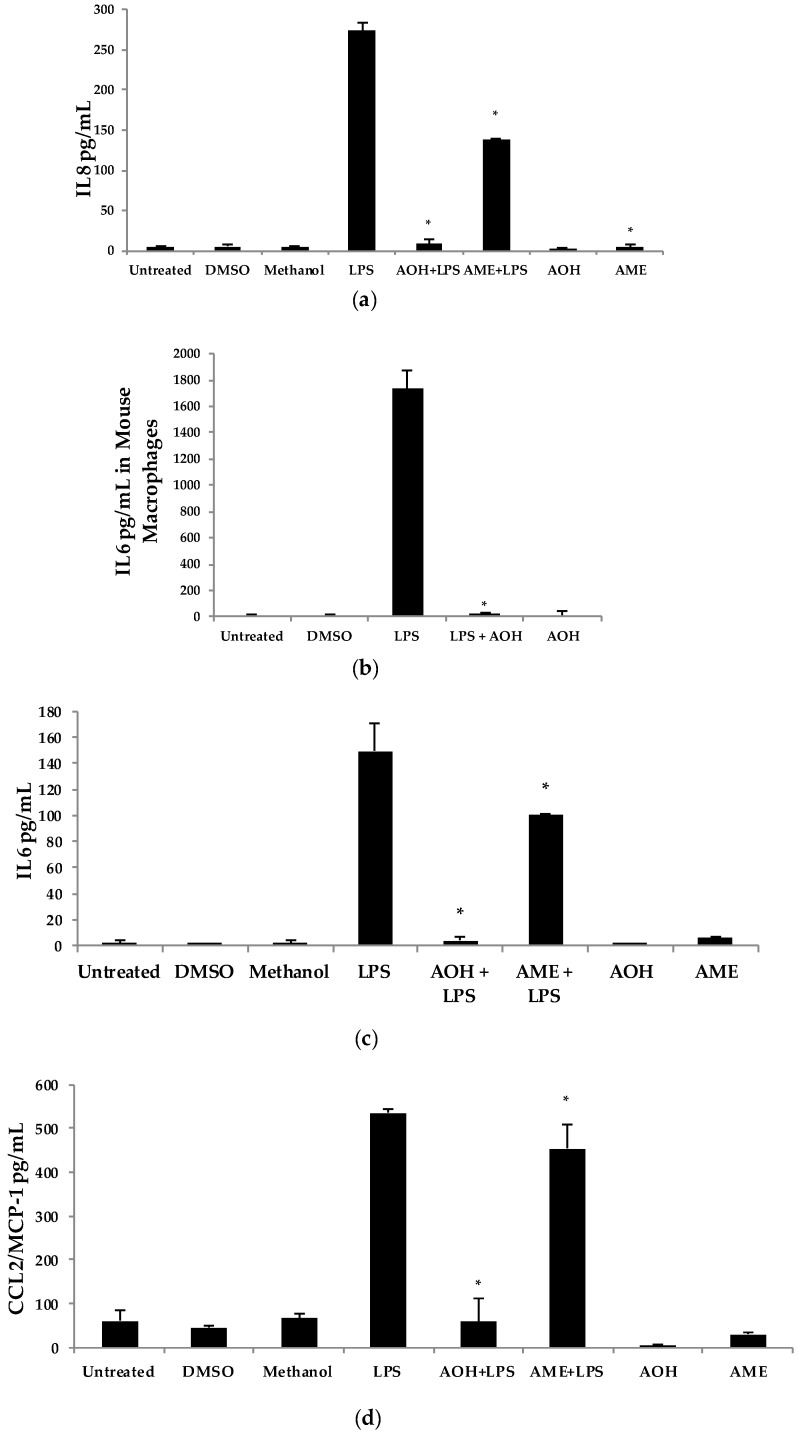
Alternariol (AOH) and alternariol monomethyl ether (AME) suppress lipopolysaccharide (LPS)-induced innate immunity. BEAS-2B airway epithelial cells (panels a, c, and d) and RAW 264.7 mouse macrophages (panel b) at a density of 5 × 10^5^ cells/well were treated with 10 μM of AOH and 10 μM of AME in the presence and absence of 10 μg of LPS and incubated for 24 h under normal conditions at 37 °C, 5% CO_2_. Supernatants were subsequently analyzed using enzyme-linked immunosorbent assay (ELISA) (**a**) IL8 BEAS-2B cells, (**b**) IL6 in RAW264.7 cells, (**c**) IL6 BEAS-2B cells, and (**d**) CCL2/MCP-1 BEAS-2B cells. An * indicates *p* < 0.05 according to Student’s *t*-test when comparing AOH/AME + LPS to LPS-induced controls. DMSO, dimethyl sulfoxide.

Upon discovering that AOH treatment resulted in the downregulation of *IL6* and *IL8* genes at the mRNA level, we next used the proinflammatory bacterial cell wall lipopolysaccharide (LPS) as an inducer of innate immunity. In the presence of 10 μM AOH and 10 μg/mL of LPS at 24 h, the levels of IL6, IL8, and MCP-1/CCL2 were reduced by several fold (Figure 2). In AME (10 μM)-treated cells, basal and LPS-induced cytokine and chemokine levels were reduced approximately half as much as compared to AOH-treated cells, leading to the conclusion that both AOH and AME have immunosuppressive properties, although AOH is the far more potent molecule of the two. We repeated this experimental design with mouse macrophage RAW264.7 cell line and observed similar results. In macrophages, LPS-induced IL6 was completely suppressed at a dose of 10 μM AOH (Figure 2).

**Figure 3:**
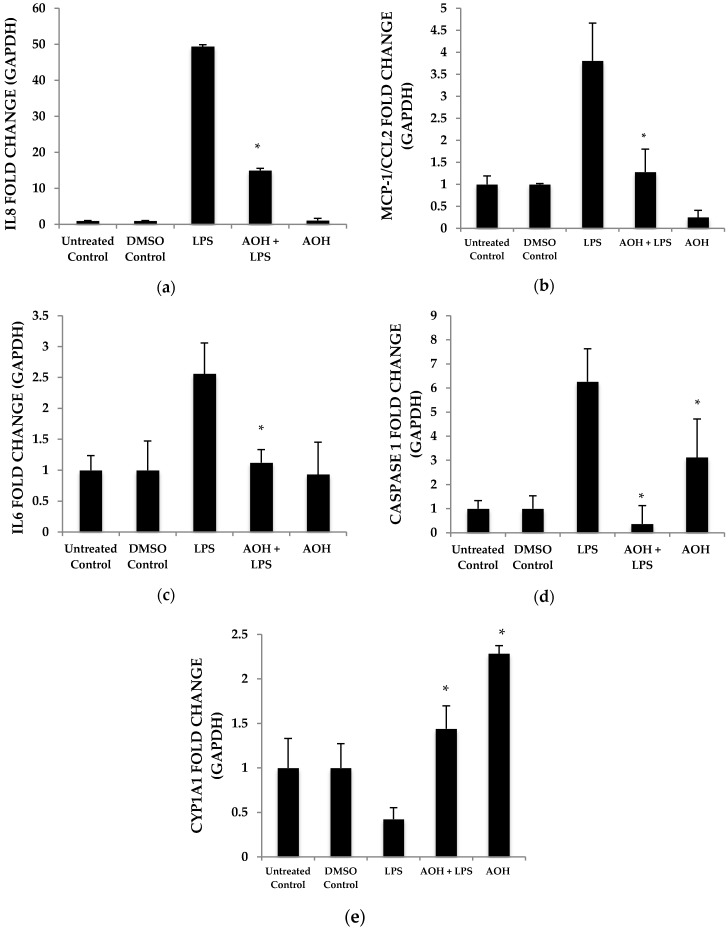
Airway epithelium treated with alternariol (AOH) and alternariol monomethyl ether (AME) results in the downregulation of LPS-induced mRNAs. BEAS-2B cells seeded at a density of 5 × 10^5^ cells/well were treated with 10 μM AOH and 10 μg LPS for 24 h. The resulting RNA was harvested and measured with quantitative real-time reverse transcription-polymerase chain reaction (qRT-PCR). Each graph here demonstrates the upregulation and downregulation (fold change) of gene expression by normalization with the control GAPDH. (**a**) IL8, (**b**) CCL2, (**c**) IL6, (**d**) Caspase 1, and (**e**) CYP1A1 fold change. An * indicates *p* < 0.05 according to Student’s *t*-test when comparing AOH to dimethyl sulfoxide (DMSO) control and AOH + LPS to LPS corresponding control.

Quantitative real-time reverse transcription-polymerase chain reaction (qRT-PCR) was used to detect changes in mRNA abundance as a measure of gene expression induced by AOH, in the presence and absence of LPS. Chemokine and cytokine gene expression profiles after normalization to the control housekeeping gene GAPDH were generated in this study for the investigation of the AOH (10 μM dose) phenotype response after a 24-h treatment in the presence and absence of 10 μg LPS. LPS-induced IL6 mRNA levels were reduced two-fold in presence of AOH. LPS-induced chemokines IL8 and MCP-1/CCL2 showed a four-fold decrease in the presence of AOH. While MCP-1/CCL2 qRT-PCR analysis showed a similar decrease of LPS-induced inflammation, it showed downregulation of the gene in the presence of AOH alone. Furthermore, we analyzed caspase 1. Caspase 1 aids in the formation of mature peptides for inflammatory cytokines interleukin-1β and interleukin-18, and is also involved in cell death and inflammasome (NLRP1 multi-molecular complex) formation [24,25]. An AOH dose of 10 μM upregulated caspase-1 but downregulated LPS-induced caspase 1 by almost five-fold, suggesting a complex mode of regulation (Figure 3). Interestingly, we observed that AOH induced cytochrome P450 CYP1A1 gene expression and partially prevented LPS-induced downregulation.

### 2.2. Dose-Dependent Analysis of Alternariol (AOH) and Lipopolysaccharide (LPS)

**Figure 4:**
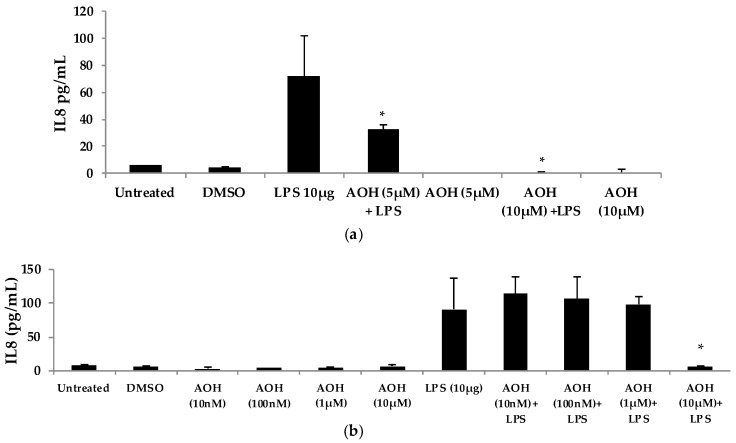
Dose-dependent response of airway epithelium cells (BEAS-2B) after treatment with alternariol (AOH) and lipopolysaccharide (LPS). (**a**) BEAS-2B cells were treated with (5–10 μM) of AOH in the presence and absence of 10 μg of LPS to measure IL8 levels released. Cell densities were 5 × 10^5^ cells/well and were incubated for 24 h under normal conditions at 37 °C, 5% CO_2_ after treatment; (**b**) BEAS-2B cells were treated with (10 nM–10 μM) of AOH in the presence and absence of 10 μg of LPS to measure IL8 levels released in supernatants using enzyme-linked immunosorbent assay (ELISA). Cell densities were 5 × 10^5^ cells/well and were incubated for 24 h under normal conditions at 37 °C, 5% CO_2_ after treatment. An * indicates *p* < 0.05 according to Student’s *t*-test when comparing AOH + LPS treatments to LPS-induced control.

We next evaluated varying doses of AOH and LPS in order to determine minimum concentrations of AOH with immunosuppressive activity. We found that AOH is highly immunosuppressive in a dose-dependent manner in both BEAS-2B and RAW264.7 cells (Figure 4). IL8 protein levels were measured in BEAS-2B cells. BEAS-2B supernatants following AOH doses of 10, 100 nM, 1, 5, and 10 μM were analyzed by enzyme-linked immunosorbent assay (ELISA). As expected, we observed an AOH dose-dependent decrease in LPS (10 μg)-induced inflammation in BEAS-2B cells. Although in all the above-mentioned doses IL8 was not detected with AOH alone, significant LPS-induced IL8 suppression was observed starting at the 5 μM dosage. A dose of 10 μM showed the highest amount of IL8 suppression. IL8 levels in LPS-induced cells treated with 10 μM AOH were equivalent or less than the levels in untreated cells (Figure 4). Similar results were observed in experiments using RAW264.7 macrophages (see Appendix A, Figure A3).

**Figure 5:**
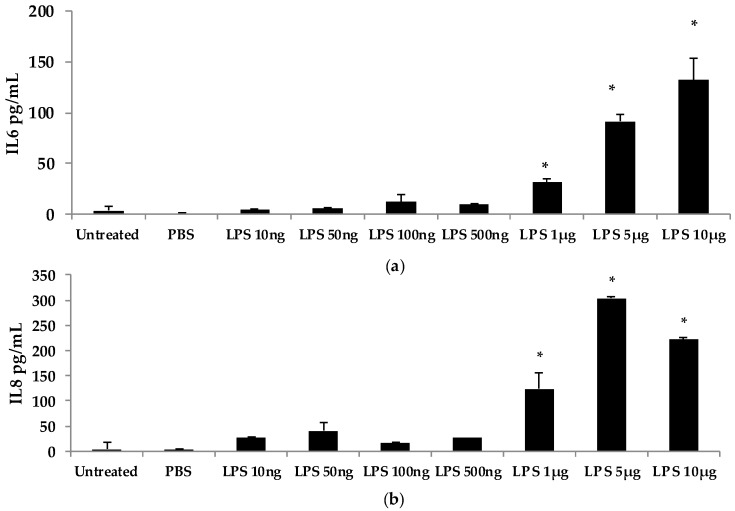
Dose-dependent response of bronchial epithelial BEAS2-B cells to LPS. LPS was added to BEAS-2B cells at a density of 500,000 cells/well for 24 h. (**a**) IL6 measured by enzyme-linked immunosorbent assay (ELISA); (**b**) IL8 measured by ELISA. An * indicates *p* < 0.05 according to Student’s *t*-test comparing individual treatments to phospho-buffered saline (PBS) control.

To further investigate the dose-dependent response of AOH, we conducted an experiment to investigate LPS doses on bronchial lung epithelial cells (BEAS-2B). Our previous experimental design of a 24-h cell treatment was applied to evaluate the protein levels of IL6 and IL8 using ELISA. We tested LPS doses including 10ng, 50 ng, 100ng, 500 ng, 1 μg, 5 μg, and 10 μg (Figure 5). A dose of 10 μg of LPS resulted in the induction of 132 pg/mL of IL6 and 221 pg/mL of IL8. With these results, we validated the doses of 10 μg of LPS and 10 μM of AOH as sufficiently substantiated for further experiments. Similar results were observed in experiments with RAW264.7 macrophages (see Appendix A, Figure A3).

**Figure A1:**
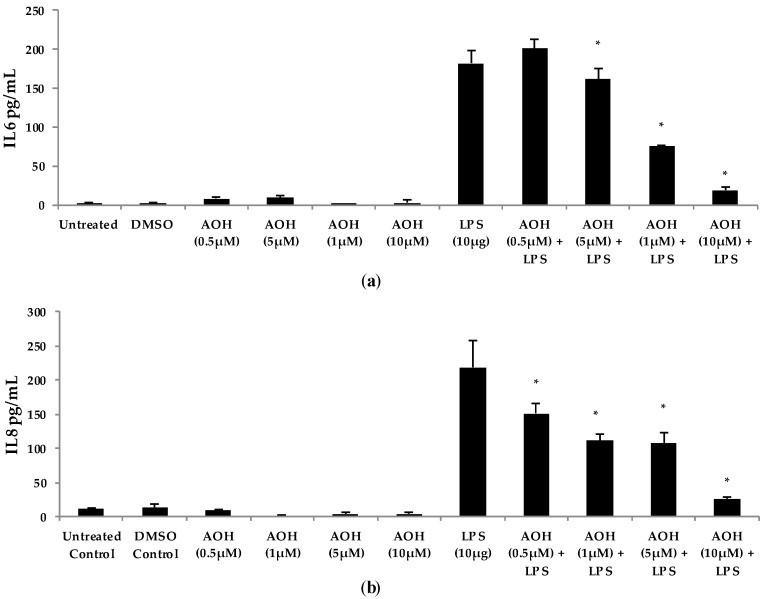
Dose-dependent analysis of the alternariol (AOH) response. Lipopolysaccharide (LPS) was added 2 h after AOH. BEAS-2B cells were seeded at a density of 500,000 cells/well. Cells were treated with 0.5–10 μM of AOH in the presence and absence of 10 μg of LPS to measure the cytokine levels released. Cell densities were 5 × 10^5^ cells/well, and they were incubated for 24 h under normal conditions at 37 °C, 5% CO_2_. Cells treated with AOH showed a marked suppression of cytokines both in the presence and absence of LPS. (**a**) IL6 measured by enzyme-linked immunosorbent assay (ELISA); (**b**) IL8 measured by ELISA. An * indicates *p* < 0.05 according to Student’s *t*-test when comparing LPS + AOH treatments to LPS-induced control.

To test whether AOH-induced immune suppression is dependent on the timing of LPS addition, we treated BEAS-2B cells with AOH and added LPS two hours later. A range of doses of AOH (10–100 μM) and AME (1–30 μM) were tested on BEAS-2B cells. All treatments resulted in a marked decrease in IL6, IL8, and MCP-1/CCL2 in the presence of LPS, indicating that AOH can prevent LPS-induced innate immune response when LPS is added after several hours (see Appendix A, Figure A1).

### 2.3. Cell Morphology Alterations in Response to AOH

**Figure 6:**
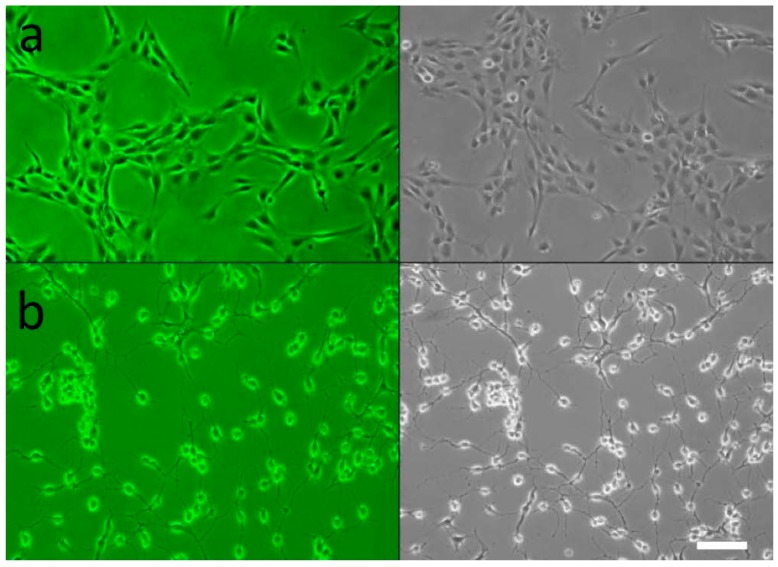
Human airway epithelial cells in the presence of alternariol (AOH). BEAS-2B cells were incubated with 10 μM of AOH for 24 h under normal conditions at 37 °C, 5% CO_2_. The images were taken with confocal microscopy with a cell density of 5 × 10^5^ cells/well (magnification 200×). (**a**) Untreated BEAS-2B cells at 24 h in color (upper left panel) and grey-scale (upper right panel); (**b**) BEAS-2B with 10 μM AOH at 24 h in color (lower left panel) and grey-scale (lower right panel). Scale bar = 100 μm.

Up until this time, no microscopic studies have been performed on AOH-treated mammalian lung epithelial cells. Multiple morphological changes were observed in the cells after treatment with AOH. BEAS-2B cells treated with AOH showed a marked change after 24 h (Figure 6). The cells demonstrated a more detached and spread out pattern of growth as well as elongated arms. This indicates that the cell cycle arrest at the G2/M phase reported in earlier studies with other cell types could be an underlying cause of cell stress observed in BEAS-2B cells, and therefore could be responsible for the change in morphology.

### 2.4. AOH Inhibits Cell Proliferation and Has Minimal Effects on Cell Death in BEAS-2B Cells

**Figure 7:**
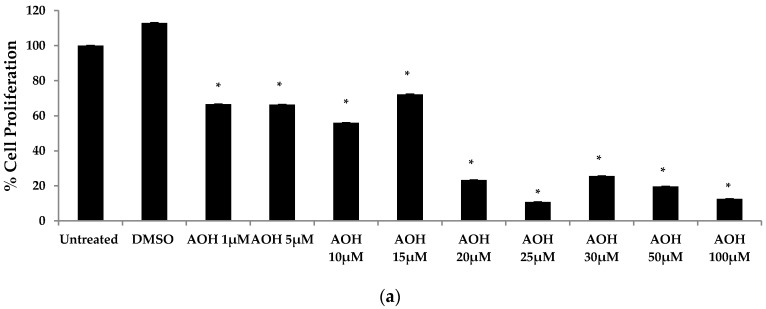

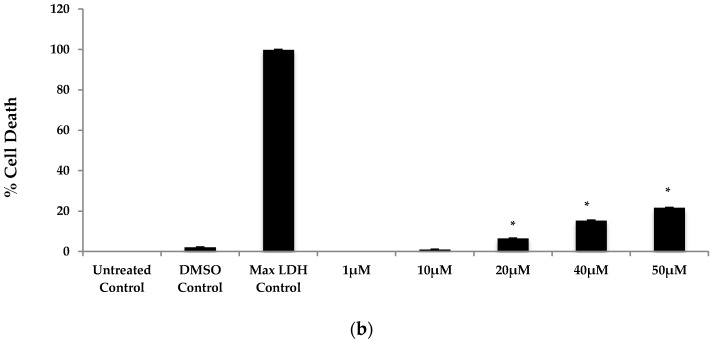
Cell proliferation and cell death analysis of BEAS-2B cells treated with alternariol (AOH). (**a**) A dose-dependent analysis of cell proliferation of BEAS-2B cells after treatment with AOH was performed by MTT assay. Cells were seeded at a density of 500,000 cells/well for 24 h; (**b**) A dose curve of lactate dehydrogenase (LDH) assay to measure the amount of LDH released by dead cells upon treatment with AOH for 24 h at a cell density of 20,000 cells/well. An * indicates *p* < 0.05 according to Student’s *t*-test for AOH treatments compared to dimethyl sulfoxide (DMSO)-treated controls.

Previous studies have emphasized the ability of AOH to cause cell death and cell cycle arrest [16,17,18,19]. Hence, a colorimetric MTT assay was performed in order to first investigate cell proliferation with doses ranging from 1 to 100 μM of AOH. In the MTT assay, the yellow MTT 3-(4,5-dimethylthiazol-2-yl)-2,5-diphenyltetrazolium bromide is reduced to purple formazan in the mitochondria of living cells. At a concentration of 10 μM of AOH, proliferation was 56% compared to control cells. It was further reduced to 23% at 20 μM and at 100 μM; only 12% of the cells were proliferating compared to controls (Figure 7).

Next, we employed a lactate dehydrogenase (LDH) assay to measure cell death. Less than 10% cell death was detected at 10 μM AOH-treated cells compared to control cells. Collectively, the MTT and LDH assay results suggest that cell cycle arrest, not cell death, is most likely responsible for the ability of AOH to suppress LPS-induced innate immune responses.

### 2.5. AOH Causes Cell Cycle Arrest in Lung Epithelium

**Figure 8:**
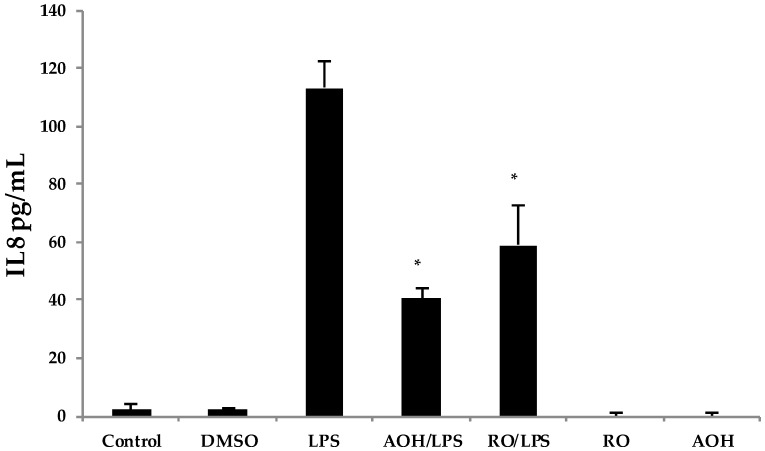
Treatment of airway epithelium cells by alternariol (AOH), RO-3306, and lipopolysaccharide (LPS). BEAS-2B cells seeded at a density of 5 × 10^5^ cells/well were treated with 10 μM of AOH or 10 μM of RO-3306 in the presence and absence of 10 μg of LPS and incubated for 24h. Supernatants were analyzed using enzyme-linked immunosorbent assay (ELISA). An * indicates *p* < 0.05 according to Student’s *t*-test for AOH/LPS and RO/LPS treatments compared to the LPS treatment alone.

The reduction in cell proliferation may be an intrinsic property of AOH, caused by the short and yet reversible arrest in the G2/M phase of the cell cycle. To further investigate whether or not cell cycle arrest may be the cause of immune suppression observed in our studies, we used the compound RO-3306, a selective ATP-competitive inhibitor of the cyclin dependent kinase, CDK1 [26]. 1 CDK1 is a serine/threonine kinase that controls the progression of cell cycle through each checkpoint (courtesy of the Cimini Lab, Virginia Tech, Blacksburg, VA, USA). RO-3306 has been identified to cause cell cycle arrest at the G2/M phase, similar to what has been reported for AOH at a dose of 10 μM [18,26]. Hence, we treated BEAS-2B cells with 10 μM AOH and 10 μM RO-3306 in the presence and absence of 10 μg LPS. We profiled the IL8 protein levels using ELISA. The data showed that RO-3306 exhibited similar immune suppressive properties as AOH (revealing a reduction of LPS-induced IL8), but was less potent. No IL8 induction was seen in cells treated with RO-3306 alone (Figure 8). These data suggest that the ability of AOH to suppress IL8 may be at least partially dependent upon its ability to cause cell cycle arrest.

### 2.6. Aryl Hydrocarbon Receptor Analysis and Mechanism of AOH-Induced Immune Suppression

**Figure 9:**
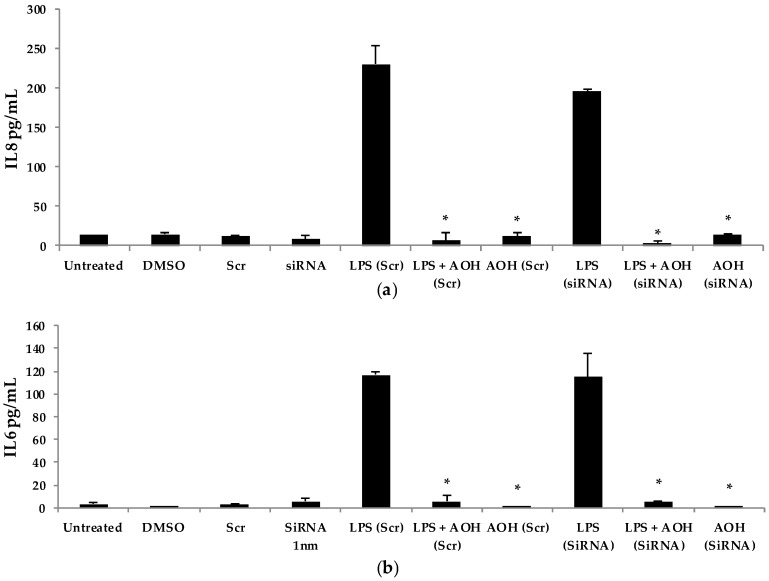
RNA silencing of the aryl hydrocarbon receptor (AhR) gene followed by treatment with lipopolysaccharide (LPS) and alternariol (AOH) in BEAS-2B cells. Cells were seeded at a density of 150,000 cells/well. Cells were treated with AhR siRNA for 24 h twice to successfully knockdown AhR. (**a**) IL8, and (**b**) IL6 released upon treatment with 10 μM AOH and 10 μg LPS for 24 h as measured by enzyme-linked immunosorbent assay (ELISA). An * indicates *p* < 0.05 according to Student’s *t*-test for AOH treatments (Scr or SiRNA) compared to LPS (Scr) or LPS (siRNA) controls, respectively.

**Figure A2:**
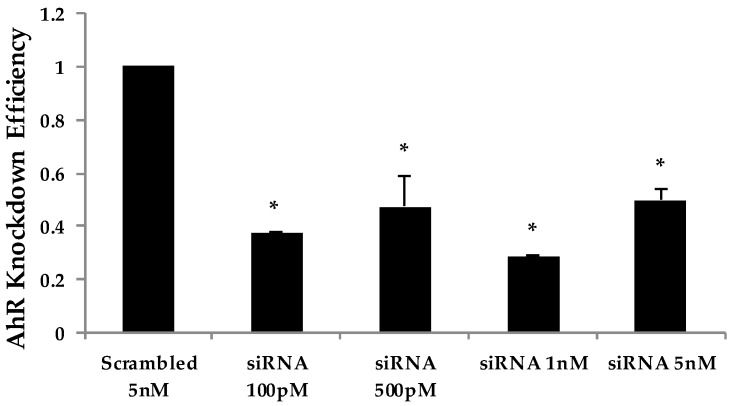
Quantitiatve real-time reverse transcription polymerase chain reaction (qRT-PCR) analysis of doses for aryl hydrocarbon receptor (AhR) silencing. Cells were seeded with a density of 150,000 cells/well. Cells were treated with AhR siRNA for 24 h. A dose curve was performed to elucidate the concentration required for AhR silencing in lung epithelial cells BEAS-2B. An * indicates *p* < 0.05 according to Student’s *t*-test for AhR-specific siRNA treatments compared to the scrambled siRNA control.

**Figure A3:**
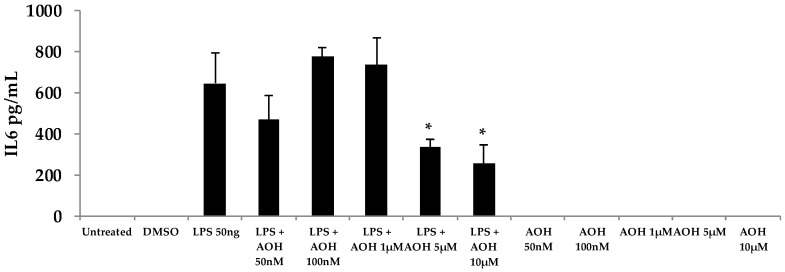
Dose-dependent response of mouse macrophages (RAW264.7) after treatment with alternariol (AOH) and lipopolysaccharide (LPS). Cells were treated with 50–10μM of AOH in the presence and absence of 50 ng of LPS to measure the IL6 levels released by enzyme-linked immunosorbent assay (ELISA). Cell densities were 5 × 10^5^ cells/well, and they were incubated for 24 h under normal conditions at 37 °C, 5% CO_2_ after treatment. An * indicates *p* < 0.05 according to Student’s *t*-test when comparing LPS + AOH treatments to LPS-induced control.

Next, we hypothesized that the aryl hydrocarbon receptor (AhR) is the target receptor for AOH that triggers downstream signaling related to its immunosuppressive properties. RNA silencing was used to knockdown the gene encoding AhR in BEAS-2B cells. Following optimization, we typically obtained a minimum of 70% knockdown of AhR using gene-specific siRNAs (see Appendix A, Figure A2). After AhR knockdown using gene-specific siRNAs, BEAS-2B cells were treated with AOH in the presence and absence of LPS for 24 h, and supernatants were then subject to ELISA. No change was observed that correlated with the silenced AhR gene, suggesting that this receptor may not be important for the ability of AOH to suppress LPS-induced immunity in BEAS-2B cells (Figure 9).

**Figure 10:**
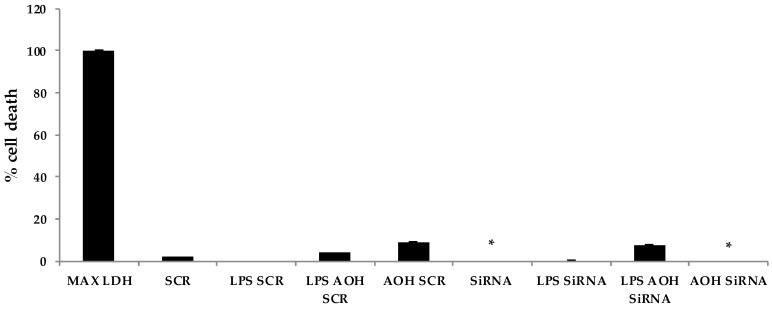
Alternariol (AOH) induced cell death is dependent upon the aryl hydrocarbon receptor (AhR). An LDH assay was performed on BEAS-2B cells with silenced AhR, 10 μM AOH, and 10 μg lipopolysaccharide (LPS). An * indicates *p* < 0.05 according to Student’s *t*-test when comparing AhR gene-specific (siRNA) treatments to their appropriate scrambled siRNA (SCR) controls.

Finally, we attempted to determine whether the modest cell death-inducing property of AOH is dependent upon AhR in BEAS-2B cells. Significant differences were detected in cell death after AOH treatments when comparing AhR silenced cells to scrambled controls, indicating that AhR may be important in causing cell death in BEAS-2B cells (Figure 10).

## 3. Discussion

This is the first study to investigate the immunomodulatory effects of AOH/AME on mammalian cells. Collectively, our data shows that AOH/AME caused a decrease in inflammatory responses in BEAS-2B bronchial epithelial cells and murine RAW264.7 macrophages when stimulated with LPS. Our data implicated that the immunosuppressive property of AOH/AME may be associated with cell cycle arrest at the G2 phase. The cell proliferation and cell death assays conducted in this study raised our understanding of the cytotoxic effects of this compound at various doses in lung epithelial cells. For example, the MTT assay results in BEAS-2B cells suggest that AOH decreased cell proliferation by almost 50% at a 10 µM dose. Along with our experiments using the G2 phase cell cycle arresting agent RO-3306, this may further implicate that the immunosuppressive properties of AOH may be related to its ability to cause cell cycle arrest. The cell death assay showed that AOH is cytotoxic to lung epithelial cells, primarily at a dose of 20 µM or higher. Results of our experiments using the 10 μM dose provides evidence that the cell death at this concentration is minimal and thus has little effect on the immunosuppressive properties of AOH. Our data using an siRNA knockdown approach also indicates that the modest cell death caused by AOH but not immunosuppression in BEAS-2B cells is most likely dependent on the AhR receptor.

In the context of allergic disease, our results suggest that AOH/AME are not plausible targets for designing therapeutics for reducing *Alternaria*-induced inflammation. In fact, the opposite may be true. Further supporting the results of this study, preliminary experiments utilizing AOH/AME-deficient *Alternaria* mutant spores have shown that they cause dramatically increased innate immune responses in BEAS-2B cells compared to wild-type fungal strains that produce normal levels of AOH/AME [27].

## 4. Materials and Methods

### 4.1. Materials

Alternariol (AOH) (Cayman Chemical, Ann Arbor, MI, USA) was reconstituted at 1 mg/mL in DMSO (Sigma-Aldrich, St. Louis, MO, USA). Alternariol monomethyl ether (AME) (Sigma-Aldrich) was reconstituted at 1 mg/mL in methanol. Ultrapure bacterial endotoxin Lipopolysaccharide (Sigma-Aldrich), cell culture grade, was reconstituted to a final concentration of 1 mg/mL in phosphate buffered saline (PBS) (Fisher Scientific, Pittsburgh, PA, USA). RO-3306 (Sigma-Aldrich) was reconstituted at 1 mg/mL in DMSO. The stock solutions were stored at −20 °C until further use.

### 4.2. Cell Culture and Cell Lines

BEAS-2B, a human bronchial lung epithelial cell line and mouse macrophage raw 264.7 cell lines were maintained in RPMI-1640 culture medium (Fisher Scientific) with 10% heat-inactivated fetal bovine serum (FBS) (Fisher Scientific) and 1% penicillin-streptomycin (ThermoFisher, Pittsburgh, PA, USA) in round bottom tissue culture-treated plates (Fisher Scientific). BEAS-2B and macrophages were incubated in 5% CO_2_ at 37 °C. Both above-mentioned cell lines were starved for 2 h before treatment with secondary metabolites in RPMI-1640 and 1% penicillin-streptomycin. BEAS-2B and macrophage cells were seeded at a density of 500,000 cells/well in 6-well tissue culture plates prior to treatment, unless indicated otherwise.

### 4.3. Quantification of Protein Levels of Cytokines and Chemokines

The cells in 6-well plates were seeded in 1.5 mL RPMI-1640 media and cells in 12-well plates were seeded in 1 mL RPMI-1640 media. BEAS-2B cells were seeded on the plates in triplicates and, after an overnight incubation at 37 °C and 5% CO_2_, washed with Dulbecco’s phosphate-buffered saline (DPBS) (Fisher Scientific). The cells were then placed in the starve media for 2 h, after which they were washed again with DPBS before being placed in fresh RPMI-1640 media. AOH, AME, and LPS were then added to the media. BEAS-2B cells were incubated for 24 h. The resulting supernatant and genetic material were collected with trypsin (Sigma-Aldrich) and stored at −80 °C. The protein levels in the cells were analyzed with enzyme-linked immunosorbent assay (ELISA) kits (Biolegend, San Diego, CA, USA) and (ThermoFisher, Pittsburgh, PA USA) following the instructions of the manufacturers. The absorbance was recorded with a microplate reader at 450 nm.

### 4.4. Quantitatification of Gene Expression

The RNA samples were isolated by applying trypsin (Sigma-Aldrich) to the AOH/AME/LPS-treated BEAS-2B or RAW264.7 cells and RNA was extracted using mammalian RNA extraction kits and manufacturer protocols (Qiagen, Valencia, CA, USA). The samples were processed into cDNA following the manufacturer’s instructions from the Bioline Tetro cDNA synthesis kit, and then stored at −20 °C (Bioline, London, UK). All the qRT-PCR reactions for the biological triplicates were performed as technical duplicates using the cDNA as a template. GAPDH was used as a control housekeeping gene for all experiments, as it has a continuous expression in mammalian cell lines. A BIO-RAD Iq5 Multicolor Real-Time PCR Detection System machine was used to conduct the qRT-PCR reaction (Bio-Rad, Hercules, CA, USA). All reactions were carried out at 20 μL volume with SYBR Green (Bioline) as the fluorescent reporter molecule. Relative fold change in gene expression was calculated using the 2(-Delta Delta C(T)) method and Pfaffl equation by normalization to GAPDH, as suggested by Bio-Rad protocols.

### 4.5. Analysis of Cell Death and Proliferation

The 3-(4,5-dimethylthiazol-2-yl) 2,5-diphenyltetrazolium bromide (MTT) solution was added to 50 μL of RPMI-1640 starve media harvested from cells treated with AOH at the 24-h time point. The plates were incubated at 37 °C for 4 h for the reduction of MTT formazan. Subsequently, 100 μL of DMSO was used to stop the reaction. Absorbance was measured at a wavelength of 570 nm using a microplate reader. The lactate dehydrogenase (LDH) assay was performed using the Pierce™ LDH Cytotoxicity Assay Kit (ThermoFisher). Cells were seeded at a density of 10,000 cells/well in 100 μL RPMI-1640 media, in 96-well flat bottom plates and incubated overnight at 37 °C and 5% CO_2_. After a 24-h treatment, 50 μL of media from each well was transferred to a new plate and 50 μL of LDH reaction mixture was added. To measure LDH activity, the reaction was stopped after a 30-min incubation and the absorbance measured at 490 nm was subtracted from the absorbance measured at 680 nm. All treatments were performed in biological triplicates and technical replicates. Percent cytotoxicity was calculated using the formula:

$$\%\;Cytotoxicity=\frac{Compound-treated\;LDH\;activity\;–\;Spontaneous\;LDH\;activity\;DH\;activity}{Maximum\;LDH\;activity\;–\;Spontaneous\;L}\times 100$$

### 4.6. Microscopy

The surface morphology of bronchial lung epithelial cells was imaged by a Nikon Eclipse TE2000-U Inverted Microscope (Nikon, Tokyo, Japan). Cells were seeded at a density of 500,000 cells/wells and treated with 10 μM AOH for 24 h before imaging.

### 4.7. RNA Silencing

Cells were seeded at a density of 125,000 cells/well. Silencing of AhR receptor was performed using a target-specific 19–25 nucleotide siRNA (Santa Cruz Biotechnology, Santa Cruz, CA, USA) designed to knockdown its gene expression. The siRNA reagent was mixed with HiPerFect Transfection Reagent (Qiagen) for transfection. BEAS-2B cells were treated with two consecutive doses of 1 nM of siRNA for 24 h to achieve a knockdown efficiency of >70%. Cells were then treated with AOH, AME, and LPS for 24 h. All experiments were performed in biological triplicates, along with a scrambled siRNA control (Santa Cruz Biotechnology). Primer pair human AHR_F and human AHR_R were used for determining knockdown efficiency.

### 4.8. Statistical Analysis

All tests were performed as biological triplicates and technical triplicates. Standard deviation was calculated from among biological replicates. The difference between individual treatment groups was validated using an unpaired Student’s *t* test for independent samples, including LPS alone and LPS stimulation in the presence of AOH. A *p*-value < 0.05 was regarded as statistically significant.

## 5. Conclusions

In conclusion, this is the first study to demonstrate that the mycotoxins AOH and AME from the fungus *Alternaria* are capable of preventing LPS-induced inflammatory responses in both a human bronchial epithelial cell line (BEAS-2B) and a mouse macrophage cell line (RAW264.7). It will be interesting in the future to further dissect the role of AOH/AME in the context of allergic inflammation in *Alternaria*-mouse models of allergy and asthma. From a broad perspective, AOH/AME may have potential and serve as the structural basis for designing new anti-inflammatory therapeutics.

## Appendix A

**Table A1:** Summary of alternariol (AOH) dosage and treatment conditions for BEAS-2B cells. No IL6 and IL8 protein level production was observed. IL6 and IL8 mRNAs were found to be downregulated upon stimulation with AOH in BEAS-2B cells.

AOH Dose	Time	IL6 (Protein)	*IL6* (Gene)	IL8 (Protein)	*IL8* (Gene)
25, 50, 100 μM	6 h	No Induction	Downregulation	No Induction	Downregulation
25, 50, 100 μM	12 h	No Induction	Downregulation	No Induction	Downregulation
25, 50, 100 μM	24 h	No Induction	Downregulation	No Induction	Downregulation

**Table A2:** Primers used in this study.

Gene Primer Name	Primer Sequence
Human AHR_F	5′ TGGTTGTGATGCCAAAGGAAG 3′
Human AHR_R	5′ GACCCAAGTCCATCGGTTGTT 3′
Human CYP1A1_F	5′ GAACCTTCCCTGATCCTTGTG 3′
Human CYP1A1_R	5′ CCCTGATTACCCAGAATACCAG 3′
Human Caspase1_F	5′ GTTCCTGGTGTTCATGTCTCA 3′
Human Caspase1_R	5′ CCTACTGAATCTTTAAACCACACC 3′
Human IL6_F	5′ GACAGCCACTCACCTCTT 3′
Human IL6_R	5′ TGTTTTCTGCCAGTGCC 3′
Human IL8_F	5′ TCCTGATTTCTGCAGCTCTG 3′
Human IL8_R	5′ GTCCACTCTCAATCACTCTCAG 3′
Human MCP-1/CCL2_F	5′ TGTCCCAAAGAAGCTGTGATC 3′
Human MCP-1/CCL2_R	5′ ATTCTTGGGTTGTGGAGTGAG 3′

## Abbreviations

AhR	Aryl hydrocarbon Receptor
ARNT	Aryl hydrocarbon Receptor Nuclear Translocator
AOH	Alternariol
AME	Alternariol Monomethyl ether
BEAS-2B	Bronchial Lung Epithelial Cells
CCL2	Chemokine (C–C motif) Ligand 2
cDNA	Complementary DNA
CDK1	Cyclin-Dependent Kinase 1
DMSO	Dimethyl Sulfoxide
DPBS	Dulbecco’s Phosphate-Buffered Saline
DNA	Deoxyribonucleic Acid
ELISA	Enzyme-Linked Immunosorbent Assay
FBS	Fetal Bovine Serum
GAPDH	Glyceraldehyde 3-Phosphate Dehydrogenase
HT29	Human Adenocarcinoma Cells
IL-1β	Interleukin-1 β
IL6	Interleukin 6
IL8	Interleukin 8
IgE	Immunoglobulin E
LDH	Lactate Dehydrogenase
LPS	Lipopolysaccharide
MTT	3-(4,5-dimethylthiazol-2-yl)-2,5-Diphenyltetrazolium Bromide
MN	Micronucleus Assay
MLC	Mouse Lymphoma Cell Line
PBS	Phosphate-Buffered Saline
RNA	Ribonucleic Acid
ROS	Reactive Oxygen Species
siRNA	Small Interfering RNA
TCDD	2,3,7,8-Tetrachlorodibenzo-p-Dioxin
TGF-β	Transforming Growth Factor β
TNF-α	Tumor Necrosis Factor α
qRT-PCR	Quantitative Real-Time Reverse Transcription-Polymerase Chain Reaction
XRE	Xenobiotic Response Element
